# The gonadal effects of diabetes

**DOI:** 10.1186/1687-9856-2013-S1-O9

**Published:** 2013-10-03

**Authors:** Ethel Codner

**Affiliations:** 1 Institute of Maternal and Child Research, School of Medicine, University of Chile, Santiago, Chile

## 

The functional reproductive alterations seen in women with type 1 diabetes (T1D) have changed as therapy has improved. Historically, patients with T1D and insufficient metabolic control exhibited a high prevalence of amenorrhea, hypogonadism, and infertility. Recent publications have shown that in spite of intensive insulin therapy, some delay in the age of thelarche, pubarche and menarche is still observed in girls with T1D. In addition, ovarian hyperandrogenism may be observed during late adolescence and an increased prevalence of hirsutism and polycystic ovarian syndrome (PCOS) has been described in adult women with T1D. These endocrine abnormalities may be related to non-physiologic insulin replacement therapy and to hyperglycemia.

Insulin is well known for its effects on carbohydrate metabolism, but this hormone also plays an important role in regulating ovarian function. Granulosa, theca and stromal ovarian cells may be affected by insulin deficiency or excess, which may be present in women with type 1 diabetes mellitus (T1D)[1]. Diabetes disrupts hypothalamic-pituitary-ovarian function, as documented by animal model studies which have helped to decipher the underlying basis of these conditions and have highlighted the variable contributions of defective leptin, insulin and kisspeptin signalling to the mechanisms of perturbed reproduction in T1D[2].

Effects of diabetes on gonadal function vary according to the age of the patient. Young girls during adrenarche exhibit an elevation of DHEAS, androstenedione, inhibin B and anti-müllerian hormone[3], an endocrine profile that is similar to the one observed in young girls in risk of developing polycystic ovarian syndrome later in life. During puberty a delay in pubertal development has been described which is followed by menstrual irregularities and hyperandrogenism during adolescence [1,4,5]. Despite these abnormalities in ovarian function, ovulatory functions is preserved in adolescents with type 1 diabetes[6]. Later in life, adult women with type 1 diabetes exhibit PCOS, polycystic ovaries at the ultrasonographic exam, menstrual irregularities and early decline in ovarian reserve[7-9].

Clearly, despite improvements in insulin therapy, T1D patients still suffer several significant clinical problems, such as pubertal delay, menstrual disturbances and hyperandrogenism which may ultimately lead to the development of PCOS in adulthood. (Fondecyt 1100123 y 1050452)

## References

[B1] Codner E Mook-Kanamori D Bazaes RA Ovarian function during puberty in girls with type 1 diabetes mellitus: response to leuprolide J Clin Endocrinol Metab 2005 90 3939 45 10.1210/jc.2005-0142 15855259

[B2] Codner E Merino PM Tena-Sempere M Female reproduction and type 1 diabetes: from mechanisms to clinical findings Hum Reprod Update 2012 18 568 85 10.1093/humupd/dms024 22709979

[B3] Codner E Iniguez G Isabel Hernandez M Elevated Anti-Mullerian Hormone (AMH) and Inhibin B levels in Prepubertal Girls with Type 1 Diabetes Mellitus Clin Endocrinol (Oxf) 2010 10.1111/j.1365-2265.2010.03887.x 21039723

[B4] Codner E Barrera A Mook-Kanamori D Ponderal gain, waist-to-hip ratio, and pubertal development in girls with type-1 diabetes mellitus Pediatr Diabetes 2004 5 182 9 10.1111/j.1399-543X.2004.00059.x 15601360

[B5] Gaete X Vivanco M Eyzaguirre FC Menstrual cycle irregularities and their relationship with HbA1c and insulin dose in adolescents with type 1 diabetes mellitus Fertil Steril 2010 94 1822 6 10.1016/j.fertnstert.2009.08.039 19796762

[B6] Codner E Eyzaguirre FC Iniguez G Ovulation rate in adolescents with type 1 diabetes mellitus Fertil Steril 2011 95 197 202 e1 10.1016/j.fertnstert.2010.10.041 21122841

[B7] Codner E Soto N Lopez P Diagnostic Criteria for Polycystic Ovary Syndrome and Ovarian Morphology in Women with Type 1 Diabetes Mellitus J Clin Endocrinol Metab 2006 91 2250 6 10.1210/jc.2006-0108 16569737

[B8] Codner E Escobar-Morreale HF Clinical review: Hyperandrogenism and polycystic ovary syndrome in women with type 1 diabetes mellitus J Clin Endocrinol Metab 2007 92 1209 16 10.1210/jc.2006-2641 17284617

[B9] Soto N Iniguez G Lopez P Anti-Mullerian hormone and inhibin B levels as markers of premature ovarian aging and transition to menopause in type 1 diabetes mellitus Hum Reprod 2009 24 2838 44 10.1093/humrep/dep276 19643804

